# Is There an Association between Diabetes and Neck and Back Pain? Results of a Case-Control Study

**DOI:** 10.3390/jcm9092867

**Published:** 2020-09-04

**Authors:** Lidiane Lima Florencio, Ana Lopez-de-Andres, Valentin Hernández-Barrera, Domingo Palacios-Ceña, César Fernández-de-las-Peñas, Rodrigo Jimenez-Garcia, Napoleon Perez-Farinos, David Carabantes-Alarcon, David Martinez-Hernandez, Romana Albaladejo-Vicente

**Affiliations:** 1 Department of Physical Therapy, Occupational Therapy, Rehabilitation and Physical Medicine, Universidad Rey Juan Carlos, Alcorcon, 28922 Madrid, Spain; lidiane.florencio@urjc.es (L.L.F.); domingo.palacios@urjc.es (D.P.-C.); cesar.fernandez@urjc.es (C.F.-d.-l.-P.); 2 Preventive Medicine and Public Health Teaching and Research Unit, Health Sciences Faculty, Rey Juan Carlos University, Alcorcón, 28922 Madrid, Spain; valentin.hernandez@urjc.es; 3 Department of Public Health & Maternal and Child Health, Faculty of Medicine, Universidad Complutense de Madrid, 28040 Madrid, Spain; rodrijim@ucm.es (R.J.-G.); dcaraban@ucm.es (D.C.-A.); dmartine@ucm.es (D.M.-H.); ralbadal.ucm@gmail.com (R.A.-V.); 4 Public Health and Psychiatry Department, Faculty of Medicine, Universidad de Malaga, 29071 Malaga, Spain; jnpf.uma@gmail.com

**Keywords:** diabetes, neck pain, low back pain, case control, predictors

## Abstract

We aimed to assess if subjects with diabetes exhibit higher prevalence of chronic back pain than age-sex-province of residence-matched non-diabetic controls. We also aimed to identify predictors for chronic neck pain (CNP) or chronic low back pain (CLBP) among subjects with diabetes. A case control study was conducted using data obtained from the Spanish National Health Survey 2017. Multivariable conditional and unconditional logistic regression models were constructed. A total of 2095 diabetes sufferers and 2095 non-diabetic matched controls were analyzed. The prevalence of CNP and CLBP was 27.3% and 34.8%, respectively, in diabetes sufferers and 22.1% and 29.0% in non-diabetes controls (both, *p* < 0.001). After multivariable analysis, the ORs showed significantly higher adjusted risk of CNP (OR 1.34; 95% CI 1.19–1.51) and CLBP (OR 1.19, 95% CI 1.09–1.31) in diabetes cases. Diabetes sufferers with CNP or CLBP showed higher use of pain medication and higher prevalence of migraine/frequent headache than controls. Female sex, worse self-rated health and use of pain medication were predictors for CNP and CLBP in subjects with diabetes. CNP and CLBP are significantly more prevalent in diabetes sufferers than in controls. Current results can help to design better preventive and educational strategies for these highly prevalent and burdensome pains among diabetic patients.

## 1. Introduction

Diabetes is a chronic disease representing a major health problem worldwide. It is a potentially disabling condition ranked as the 18th most prevalent non-fatal condition globally [1]. Most concerning data is that current rates of prevalence and years of living with disability is still worsening [1,2,3]. Furthermore, projections from 2016 to 2040 demonstrated that diabetes can evolve from the 15th to the 7th cause of premature mortality [4]. Similarly, the global economic burden of diabetes could increase substantially even when considering the best international target scenario [5].

Besides global estimations, regional characteristics should always be considered. In Spain, from 1990 to 2016, diabetes moved down from 6th to 10th cause of death but it was maintained the 9th in the rank among the most disabling conditions considering the years living with disability [6]. The challenge to the health system and health care providers is not the only the diabetes itself but also its related complications and comorbidities [5,6,7,8]. Chronic musculoskeletal pain is a frequent comorbid condition of diabetes [9,10,11]. Similar to diabetes, low back and neck pain also represent prevalent and disabling conditions which have shown a 19% increase in rate of prevalence and years living with disability from 2005 to 2015 [12]. Data from the 2017 Global Burden Disease revealed that low back pain was ranked as the first, diabetes as the 4th and neck pain as the 9th for females and 11th for males most disabling conditions worldwide [1]. In Spain, neck and low back pain are ranked as the first [6]. Moreover, the individual burden of having comorbid diabetes to musculoskeletal pain is substantially greater than the burden of having just one of each condition [13,14,15].

The Spanish National Healthcare System guarantees universal coverage and free healthcare access to all Spanish nationals, regardless of economic situation or participation in the social security network and is principally funded through taxation.

Some factors associated with the coexistence of diabetes and musculoskeletal pain include female sex, greater BMI, sedentary lifestyle, impaired physical function, mental health disorders and medication intake [16,17].

Previous studies have found that diabetes mellitus may be a predisposing factor for the development of lumbar spinal stenosis [18]. The effect of diabetes on the immunological system could result in the development of secondary infections in the back-causing LBP [19]. The presence of diabetes may alter the properties of white matter and this can be a cause, predisposing factor, consequence or compensatory adaptation of chronic musculoskeletal pain [20].

Even if several previous studies have researched diabetes and spinal pain the differences in methodologies and settings (health centers or population based), variables collected, small sample sizes and statistical methods make comparisons difficult and conclusions dubious [9,10,11,13,15,16,17,18].

Better understanding of potential association between diabetes and musculoskeletal pain in the spine and identification of attributable risk factors are key components of public health policies enabling better management of the patients in order to reduce their morbidity [6,17]. Therefore, the current study aimed to assess if subjects with diabetes exhibit higher prevalence of chronic back pain (neck and low back pain) than age-sex-province of residence-matched non-diabetic controls using the Spanish National Health Survey conducted in 2017 (SNHS2017). We also aimed to identify the variables associated with suffering from chronic neck pain (CNP) or chronic low back pain (CLBP) among subjects with diabetes.

## 2. Materials and Methods

### 2.1. Design, Setting and Participants

A case control study was conducted using the data obtained from the SNHS2017 conducted in Spain from October 2016 to October 2017. Details in the SNHS2017 methodology can be found elsewhere [21]. Briefly, the SNHS2017 includes a representative sample of the Spanish Population aged 15 years or over residing in main family dwellings. The sampling method is a stratified three-stage sampling, with the first stage units being the census tracts, the second stage the main family dwellings, and in the third stage an adult (aged ≥15 years old) randomly selected (Kish method) within each household to fill in the used questionnaire [21]. The information collection method is the computer-assisted personal interview (CAPI).

### 2.2. Main Outcomes Measures

In the SNHS2017, self-reported presence of chronic conditions was collected using the following three consecutive questions: 1. Do you have or have you ever had any of the following diseases or heath conditions? 2. Have you suffered this disease/health condition within the past 12 months? and 3. Was this disease/health condition diagnosed by a doctor? A card with a list of 32 conditions was shown to the person interviewed after the first question and, for those conditions reported by the participant, the second and third questions were completed consecutively for each specific condition.

Participants were asked if they suffered diabetes and only persons who answered affirmatively to the three questions used to identify its presence were considered cases as “diabetes sufferers”. Those subjects interviewed that answered “yes” only the first or to the first two questions and “no” to the third question were excluded from the study sample, so they could not be selected as a control.

The same method was used to identify participants who suffered CNP or CLBP. Participants were informed by the interviewer, prior to answering these questions, that a “chronic disease or health condition” is that one lasting for at least six months [21].

The independent variables are analyzed, and their categories are detailed in Table 1 and Table 2 and included, gender, age, educational level, living with a partner, self-rated health and limitations for usual activities. The pain characteristics analyzed were pain intensity and use of pain medication prescribed by a physician. The information regarding pain intensity was obtained with the question “Over the last four week, what intensity of pain have you suffered?” Six possible options were given: (1) None, (2) Very light, (3) Light, (4) Moderate, (5) Severe and (6) Extreme. For study purpose we grouped these options in three categories “Light” (including “very light” and “light”), “Moderate” (including “moderate”) and “Severe/extreme” (including “severe” and “extreme”) [22].

The same method (questions) used to identify diabetes, CNP or CLBP was applied to determine the presence of asthma, chronic obstructive pulmonary disease (COPD), heart disease, stroke, cancer, high blood pressure mental disorders (anxiety/depression) and a migraine/frequent headache. Lifestyle variables included body mass index, physical exercise, tobacco use and alcohol consumption.

All questions included in the SNHS2017 and explanations on how the questionnaires are conducted are described elsewhere [21,22].

### 2.3. Matched Case Control Design

The mean age of individuals aged 15 years or over in the SNHS2017 with self-reported physician diagnosis diabetes was 67.8 years (SD 13.25). The mean age of those without self-reporting diabetes was significantly lower 46.9 (SD 18.35) years (*p* < 0.001). Given this significant difference, we matched the sample of diabetes sufferers with a matched sample of non-diabetes subjects. Therefore, for each diabetes sufferer (case), we selected an age and gender-matched subject without diabetes (control) living in the same province. If more than one matched control was available for a case, the selection was randomly conducted. With this process, we were able to find a matched control for each of the 2095 diabetes sufferers included in the SNHS2017.

### 2.4. Statistical Analysis

Results are shown and compared according to the presence of diabetes. We estimated the prevalence of CNP and CLBP by the study variables. As descriptive statistics for quantitate variables, we used mean and standard deviation, and for qualitative variables, we calculated proportions. The McNemar test and paired Student t-tests were conducted to compare proportions and means between subjects with and without diabetes. To assess the association between the study variables and the presence of CNP or CLBP within the diabetic population we used the Chi square test.

Multivariable conditional logistic regression models were constructed to estimate the risk of suffering CNP or CLBP among diabetes sufferers versus no-diabetes controls after adjusting for potential confounders. Unconditional logistic regression models were used to identify which variables were independently associated with the presence of CNP and CLBP among diabetes sufferers. These models were the construction of the logistic regression model and were done following the recommendation of Hosmer et al. [23]: (i) bivariate analysis of each single variable; (ii) we included all the variables significant in the bivariate and those we considered important in the references reviewed; (iii) to fit of the multivariate model we used the Wald statistic for each variable to see its contribution to the model; (iv) the Likelihood Ration test was used to compare the new model with the previous; (v) once the final model was constructed, we checked for the existence of linearity and interactions in the model. No significant interactions were found. Adjusted Odds Ratio (OR) with 95% confidence intervals (95% CI) is the measure of association provided by the multivariable models.

The STATA 14.0 (StataCorp. 2015. Stata Statistical Software: Release 14. StataCorp LP., College Station, TX, USA) was used for statistical analysis and significance was set at two-tailed α < 0.05.

### 2.5. Ethical Aspects

In accordance with the Spanish legislation, as we used a public access dataset with anonymous data, the approval of an ethics committee is not needed. The database can be downloaded freely by anyone [24].

## 3. Results

A total of 2095 diabetes sufferers and 2095 non-diabetic matched controls were included. The distribution of the study populations is summarized in Table 1. Diabetes sufferers have a lower educational level, worse self-rated health and more limitations for usual activities. Diabetes sufferers reported a higher prevalence of all medical conditions (all, *p* < 0.01) except for cancer and migraine/frequent headache. Obesity (BMI ≥ 30) was more prevalent (33.8% vs. 20.8%; *p* < 0.001) and alcohol consumption was lower (30.3% vs. 38.5%; *p* < 0.001) in diabetes sufferers. Severe/extreme pain intensity and use of pain medication were also more prevalent in subjects with diabetes (*p* < 0.001).

The prevalence of CNP and CLBP was 27.3% and 34.8%, respectively, in diabetes sufferers and 22.1% and 29.0% in non-diabetes controls (both, *p* < 0.001). In addition, 21.1% diabetes cases and 16.6% controls reported suffering both neck and low back pain (*p* < 0.001). After multivariable analysis, the ORs showed significantly higher adjusted risk of CNP (OR 1.34; 95% CI 1.19–1.51) and CLBP (OR 1.19, 95% CI 1.09–1.31) in diabetes cases when compared with matched non-diabetic controls. This means that after adjusting by possible confounders diabetes sufferers had 34% and 19% higher adjusted risk of CNP and CLBP than non-diabetic controls.

Table 2 summarizes the prevalence of CNP and CLBP in diabetes and non-diabetes subjects according to socio-demographic variables and pain features. The prevalence of CNP and CLBP was significantly higher in diabetes sufferers than among non-diabetes controls according to all sociodemographic variables.

Among diabetes sufferers, the prevalence of CNP was more than twice as high in females than in males (37.5% vs. 16.9%; *p* < 0.001) and increased with age, from 21.5% in the youngest group to 32.0% in the oldest group. For CLBP, 44.4% of females with diabetes reported CLBP compared with 25.1% of diabetic males (*p* < 0.001). The highest prevalence of CLBP among diabetes sufferers was found in the oldest group (37.6%) vs. the lowest was found within the youngest group (23.2%). Regarding pain characteristics, diabetes sufferers with CNP or CLBP showed higher use of pain medication and also higher prevalence of migraine/frequent headache than non-diabetic controls.

As it can be observed in Table 3, the presence of CNP or LBP was associated with worse self-rated health and more limitations for usual activities in individuals with and without diabetes (*p* < 0.01), being these differences more acute in CLBP. In diabetes sufferers, the prevalence of CNP was higher in those also suffering concomitant medical conditions, particularly mental disorders (48.2%), asthma (45.6%), and COPD (40.7%). Similarly, diabetes sufferers suffering from CLBP showed more comorbidity with most medical conditions, mostly asthma (57.6%), mental disorders (55.3%), COPD (52.8%) and cancer (51.9%). Regarding lifestyle habits, a BMI > 30 and practicing less physical activity were associated with higher prevalence of CNP and CLBP in diabetes sufferers and also matched non-diabetic controls.

The predictors for suffering CNP and CLBP in diabetes sufferers after multivariable adjustment are shown in Table 4. Women showed higher probability of reporting CNP (OR 1.79, 95% 1.32–2.42) and CLBP (OR 1.37, 95% 1.01–1.86). Additionally, CNP and CLBP were associated with higher probability of reporting fair/poor/very poor self-rated health (OR 1.69, 95% CI 1.16–2.47 and 2.24, 95% 1.62–3.11, respectively) and higher pain medication use (OR 2.06, 95% CI 1.49–2.82 and 2.00, 95% 1.50–2.67, respectively). The presence of CNP or CLBP was also associated with higher probability of reporting a migraine/frequent headache. Finally, diabetes sufferers with concomitant CNP, but not those with CLBP, had higher probability of also presenting mental disorders (OR 1.48, 95% CI 1.04–2.13).

Diabetes sufferers with concomitant CLBP were significantly older (OR 1.87, 95% CI 1.37–2.58), reported more limitations for usual activities (OR 1.56, 95% CI 1.11–2.18) and had a BMI ≥ 30 (OR 1.33, 95% CI 1.17–1.53). These associations were not seen in diabetes sufferers with concomitant CNP.

Finally, the presence of concomitant CNP represented an increased risk (OR 10.46, 95% 7.72–14.17) of suffering concomitant CLBP in our sample of diabetes sufferers.

## 4. Discussion

The current population-based study revealed a greater prevalence of CNP, CLBP and the combination of both conditions in subjects with diabetes when compared to age- and sex-matched non-diabetic controls. Common factors identified within diabetes sufferers associated to the presence of CNP and CLBP included female sex, worse self-rated health status, higher use of pain medication and another concomitant disease. However, some variables were associated with a greater risk of either CNP or CLBP. The presence of mental disorders was an associated risk factor for CNP, while older age, limitation for usual activities and greater BMI were associated with a greater risk to report CLBP.

The association between diabetes and spinal musculoskeletal pain, i.e., CLBP or CNP, is in agreement with previous reports [9,10,11,16,17]. The magnitude of the association with diabetes was slightly greater for CNP (OR: 1.34) than the magnitude observed for CLBP (OR: 1.19). This is the opposite of pooled data reported in the recent metanalysis conducted by Pozzobon et al., [10] who reported an OR 1.35 for the association between diabetes and CLBP and an OR 1.24 for the association between diabetes and CNP. This discrepancy could be a reflection of regional differences related to health care policies, cultural, environment and genetics aspects that are not evident in metanalysis that pool data from different countries. On the other hand, a previous populational-based study in Spain also reported higher association between diabetes with CLBP than with CNP [17]. A change in these associations reinforces the idea that research about comorbid conditions need to be constantly updated as it can be influenced not only be regional aspects but may also be a dynamic relationship and change over the years.

The comorbid relationship between chronic back pain (neck and low back pain) and diabetes is not completely clarified. Different hypotheses have been proposed with a possible bidirectional influence. Pathoanatomical changes of the spine have been linked as a consequence of the hyperglycemia and altered fat metabolism commonly present in diabetes [25,26,27], and changes in health style and diet habits associated with chronic pain can lead to type 2 diabetes [28,29]. In fact, current literature tends to support the hypothesis that these conditions share common risk factors, such as obesity and less physical activity, instead of a causal relationship, as no causality could be determined in the longitudinal studies previously conducted [10,30,31,32,33].

An alternative explanation that could contribute to the association between diabetes and chronic back pain is what has been called the “double-crush syndrome” [34]. The metabolic alterations caused by diabetes, such as prolonged hyperglycemia, can hit the peripheral nerve first, which then becomes more susceptible to a “second hit”, by the local factors related to entrapment, such as increased pressure, strain and/or elongation in the anatomically narrow sites [34,35].

Regardless of the underlying mechanisms leading to this comorbid relationship, our data reinforce that co-existence of diabetes and spinal pain accentuates the prevalence of most severe pain intensity, more use of pain medications and other comorbid diseases (e.g., migraine, asthma, COPD or mental disorders). Additionally, the co-existence of these conditions was also associated with lower practice of physical activity and worse self-rated health perception. A negative impact of these conditions in physical activity, health-perception and quality of life has already been reported [9,16,36]. Therefore, it would be reasonable to suggest that direct and indirect burden related to diabetes will be higher if it is associated with CNP or CLBP.

Better understanding of potential associated risk factors can help to guide some strategies to manage and reduce their impact. Among those common factors associated with CNP and CLBP in subjects with diabetes, the greatest risk to report pain in one area was the presence of pain in the other site. This association is expected and is described in patients with chronic musculoskeletal pain and emphasizes that the best way to reduce the incidence of chronic pain is to prevent acute pain and proper management when it occurs [37]. We observed that consumption of pain medication was twice in subjects with diabetes and concomitant CNP or CLBP. It would be relevant to determine if alternative interventions, such as pain education and non-pharmacological management, would be more suitable options to manage comorbid conditions. For instance, implementation of regular exercise programs can be of interest for this population. In fact, exercise does not only alleviate musculoskeletal pain but also promotes improved quality of life, and reduces mortality in individuals with diabetes [38,39]. However, caution should be taken for proper and gradual prescription in people with chronic pain since they present distinct responses and adaptations to exercise [40].

The association between female sex and the greater risk to report CNP or CLBP is consistent with previous reports in patients with diabetes [16,33], and it is also consistent in chronic musculoskeletal pain [37]. Gender differences may be driven by biological and social aspects in relation to the pain experience [41,42]. However, lifestyle habits that are also recognized as risk factors for CLBP and CNP in a general population, such as alcohol and tobacco consumption [43,44], did not appear among potential risk factors in our sample of diabetes sufferers.

Finally, some variables were associated with only CNP or CLBP in diabetes sufferers. A greater BMI was a risk factor just for CLBP, but not for CNP. This finding has been previously observed in patients with diabetes [16,17]. The remained risk factors for just CLBP, e.g., older age and limitation for usual activities [44,45,46,47], and for just CNP, such as mental disorders [44,48,49], have been also associated in previous studies with these pain conditions, but without considering the presence of diabetes. Therefore, public health programs and tailored individual management of people with diabetes should consider these differences.

The strengths of this population-based study include a case-control matched design, the use of standardized surveys and training of the data collectors. Furthermore, the novelty of our work is that we analyzed sociodemographic variables that are not collected in clinical records and self-reported lifestyles and pain characteristics, variables that are not usually collected with standardized methods in the clinical settings. Furthermore, we have a large (over 2000 subjects) representative sample of the entire Spanish population suffering diabetes, not a selected sample from one or several hospitals or primary care centers. On the other hand, the current study also presents some limitations. First, there are difficulties in examining prevalence due to precise definitions of the conditions of interest. For the question related to diabetes, a study performed in Spain reported specificities >95% and sensitivities >70% using medical records as the gold standard [50]. However, it is not the case of the questions used for CNP and CLBP, since they have not been validated yet. Second, we did not assess specific characteristics of diabetes (type, duration, treatment and complications) neither did we assess the severity, duration or related-disability of CNP and CLBP which would provide further knowledge about their associations. Third, comorbid diabetes and CLBP/CNP can have various causal links (i.e., depression, discopathy, inflammation, overweight/obesity, neuropathy) and consequences (i.e., reduction in physical activity, therapy compliance, quality of life), which, given the limited information collected by the SNHS2017, could not be fully investigated. Further studies should include these relevant variables. Forth, responses were based on individual’s self-reporting (although with a medical diagnosis confirmation) so under-reporting/over-reporting may appear from recall or social desirability biases [36,37]. Fifth, the final non-response rate for the SNHS17 was 27.8%, so the existence of a non-response bias must be considered [21]. Sixth, given the cross-sectional design is not possible to discard the existence of a reverse casualty bias. Seventh, we analyzed information on physical exercise by asking the days per week the interviewed person walked for at least 10 min; these data were collected using questions included in the short version of the adapted International Physical Activity Questionnaire. [51]. The SNHS2017 measures walking to quantify the volume of physical activity in order to be able to identify the population that does not meet the World Health Organization’s recommendations on physical activity [21].

## 5. Conclusions

We conclude that diabetes was shown to be associated with a higher risk of reporting CNP and CLBP. Female sex, worse self-rated health, use of pain medication and other concomitant chronic pain were associated with greater risk of presenting CNP and CLBP among individuals with diabetes. Mental disorder was an associated risk factor only for CNP, while older age, limitation for usual activities and greater BMI were only associated with a greater risk to report CLBP. Current results can help to design better preventive and educational management strategies for these chronic conditions that are highly prevalent and burdensome among diabetic patients.

## Figures and Tables

**Table 1:** Differences in study variables among adults with diabetes and age–sex–province of residence matched non-diabetic subjects.

Variable	Categories	No Diabetes		Diabetes		*p*
		*n*	%	*n*	%	
Gender	Female	1056	50.4	1056	50.4	NA
Age groups	15–59 years	233	11.1	233	11.1	NA
	60–69 years	1093	52.2	1093	52.2	
	70 years or over	769	36.7	769	36.7	
Educational level	No studies/primary	1474	70.4	1668	79.6	<0.001
	Secondary	255	12.2	222	10.6	
	High education	366	17.5	205	9.8	
Living with a partner	Yes	1176	56.1	1163	55.5	0.686
Self-rated health	Fair/poor/very poor	1007	48.1	1397	66.7	<0.001
	Very good/good	1088	51.9	698	33.3	
Limitations for usual activities ^a^	Yes	877	41.9	1173	56.0	<0.001
Asthma	Yes	88	4.2	158	7.5	<0.001
COPD	Yes	154	7.4	214	10.	<0.001
Heart disease ^b^	Yes	273	13.0	423	20.2	<0.001
Stroke	Yes	26	1.2	51	2.4	0.004
Cancer	Yes	75	3.6	81	3.9	0.624
High blood pressure	Yes	837	40.0	1296	61.	<0.001
Mental disorders ^c^	Yes	294	14.0	396	18.9	<0.001
Migraine or frequent headache	Yes	156	7.4	185	8.8	0.101
Body mass index ^d^	<25	770	36.8	551	26.3	<0.001
	25–29.9	886	42.4	834	39.9	
	≥30	434	20.8	707	33.8	
Physical exercise ^e^	0–3 days	312	32.8	323	34.0	0.593
	4–7 days	638	67.2	627	66.0	
Tobacco use	Never	1091	52.2	1059	50.6	0.168
	Ex-smoker	664	31.8	721	34.4	
	Current smoker	336	16.1	314	15.0	
Alcohol consumption ^f^	Yes	804	38.5	635	30.3	<0.001
Pain intensity ^g^	Light	506	42.8	512	36.5	<0.001
	Moderate	423	35.8	486	34.6	
	Severe/extreme	252	21.3	405	28.9	
Use of pain medication ^h^	Yes	776	44.5	981	47.6	0.052
Chronic neck pain	Yes	464	22.1	572	27.3	<0.001
Chronic low back pain	Yes	607	29.0	730	34.8	<0.001
Chronic neck and low back pains	Yes	348	16.6	443	21.1	<0.001

NA. Not adequate as this a matching variable. ^a^ Limited because of a health problem in activities people usually do over the last 6 months. ^b^ Heart disease included coronary disease. Myocardial infarction and angina. ^c^ Mental disorder included anxiety and/or depression. ^d^ Body mass index bases on self-reported height and weight. ^e^ Physical exercise. Days per week with walking for at least 10 min. ^f^ Alcohol consumption. If the subject has consumed alcohol two or more times a month over the last year. ^g^ Pain intensity in the last 4 weeks. ^h^ Consumption of physician prescribed pain medication in last 2 weeks.

**Table 2:** Prevalence of chronic neck pain and chronic low back pain among subjects with diabetes and non-diabetic controls according to socio-demographic and pain characteristics.

		Chronic Neck Pain				Chronic Low Back Pain			
		No Diabetes		Diabetes		No Diabetes		Diabetes	
		*n*	%	*n*	%	*n*	%	*n*	%
Gender ^c,d^	Male ^a,b^	152	14.6	176	16.9	226	21.8	261	25.1
	Female ^a,b^	312	29.5	396	37.5	381	36.1	469	44.4
Age groups ^c,d^	15–59 years ^a,b^	29	12.4	50	21.5	33	14.2	54	23.2
	60–69 years ^a,b^	222	20.3	276	25.3	304	27.8	387	35.4
	70 years or over ^a,b^	213	27.7	246	32.0	270	35.1	289	37.6
Educational level ^c,d^	No studies/primary ^a,b^	365	24.8	490	29.4	473	32.1	618	37.1
	Secondary ^a,b^	47	18.4	39	17.6	56	22.0	60	27.0
	High education ^a,b^	52	14.2	43	21.0	78	21.3	52	25.4
Living with a partner ^c,d^	No ^a,b^	233	25.4	292	31.3	307	33.4	341	36.6
	Yes ^a,b^	231	19.6	280	24.1	300	25.5	389	33.4
Concomitant chronic neck pain ^c,d^	No ^b^	NA	-	NA	-	259	15.9	287	18.8
	Yes ^b^	NA	-	NA	-	348	75.0	443	77.4
Concomitant chronic low back pain ^c,d^	No	116	7.8	129	9.5	NA	-	NA	-
	Yes ^a^	348	57.3	443	60.7	NA	-	NA	-
Pain intensity ^c,d^	Light	100	19.8	101	19.7	143	28.3	140	27.3
	Moderate	158	37.4	179	36.8	213	50.4	236	48.6
	Severe/extreme	146	57.9	232	57.3	167	66.3	271	66.9
Use of pain medication ^c,d^	No	132	13.6	153	14.2	186	19.2	205	19.0
	Yes ^a,b^	313	40.3	413	42.1	393	50.6	518	52.8
Migraine or frequent headache ^c,d^	No ^a,b^	388	20.0	464	24.3	521	26.9	610	31.9
	Yes ^a,b^	76	48.7	108	58.4	86	55.1	120	64.9

^a^ Significant differences between diabetes sufferers and non-diabetes controls with chronic neck pain. ^b^ Significant differences between diabetes sufferers and non-diabetes controls with chronic low back pain. Comparisons conducted using the McNemar test. ^c^ Significant association between the variable and chronic neck pain. ^d^ Significant association between the variable and chronic low back pain. Comparisons conducted using chi square test.

**Table 3:** Prevalence of chronic neck pain and chronic low back pain among subjects with diabetes and non-diabetic controls according to health status and lifestyles variables.

		Chronic Neck Pain				Chronic Low Back Pain			
		No Diabetes		Diabetes		No Diabetes		Diabetes	
		*n*	%	*n*	%	*n*	%	*n*	%
Self-rated health ^c,d^	Fair/poor/very poor	355	35.3	480	34.4	454	45.1	617	44.2
	Very good/good ^a,b^	109	10.0	92	13.2	153	14.1	113	16.2
Limitations for usual activities ^c,d^	No ^a,b^	140	11.5	140	15.2	209	17.2	182	19.7
	Yes	324	36.9	432	36.8	398	45.4	548	46.7
Asthma ^c,d^	No ^a,b^	440	21.9	500	25.8	579	28.8	639	33.0
	Yes ^a,b^	24	27.3	72	45.6	28	31.8	91	57.6
COPD ^c,d^	No ^a,b^	411	21.2	485	25.8	547	28.2	617	32.8
	Yes ^a,b^	53	34.4	87	40.7	60	39.0	113	52.8
Heart disease ^c,d^	No ^a,b^	379	20.8	426	25.5	508	27.9	546	32.7
	Yes ^a,b^	85	31.1	146	34.5	99	36.3	184	43.5
Stroke	No ^a,b^	456	22.0	556	27.2	596	28.8	711	34.8
	Yes ^b^	8	30.8	16	31.4	11	42.3	19	37.3
Cancer ^c,d^	No ^a,b^	441	21.8	541	26.9	583	28.9	688	34.2
	Yes ^a,b^	23	30.7	31	38.3	24	32.0	42	51.9
High blood pressure ^c,d^	No ^a,b^	240	19.1	192	24.0	308	24.5	234	29.3
	Yes ^a,b^	224	26.8	380	29.3	299	35.7	496	38.3
Mental disorders ^c,d^	No ^a,b^	327	18.2	381	22.4	449	24.9	511	30.1
	Yes ^a,b^	137	46.6	191	48.2	158	53.7	219	55.3
Body mass index ^c,d^	<25 ^a,b^	159	20.6	154	27.9	188	24.4	186	33.8
	25–29.9 ^a,b^	190	21.4	193	23.1	262	29.6	261	31.3
	≥30 ^a,b^	113	26.0	225	31.8	156	35.9	283	40.1
Physical exercise ^c,d^	0–3 days ^a,b^	63	20.2	83	25.7	84	26.9	116	35.9
	4–7 days ^a,b^	104	16.3	140	22.3	137	21.5	182	29.0
Tobacco use ^c,d^	Never ^a,b^	282	25.8	342	32.3	363	33.3	418	39.5
	Ex-smoker ^a,b^	126	19.0	161	22.3	169	25.5	208	28.8
	Current smoker ^a,b^	55	16.4	69	22.0	74	22.0	104	33.1
Alcohol consumption ^c,d^	No ^a,b^	313	24.3	452	31.0	420	32.6	556	38.1
	Yes ^b^	150	18.7	119	18.7	186	23.1	172	27.1

^a^ Significant differences between diabetes sufferers and non-diabetes controls with chronic neck pain. ^b^ Significant differences between diabetes sufferers and non-diabetes controls with chronic low back pain. Comparisons conducted using the McNemar test. ^c^ Significant association between the variable and chronic neck pain. ^d^ Significant association between the variable and chronic low back pain. Comparisons conducted using chi square test.

**Table 4:** Factors associated with suffering chronic neck pain and chronic low back pain among diabetes sufferers. Results of multivariable logistic regression analysis.

		Chronic Neck Pain	Chronic Low Back Pain
		OR (95% CI)	OR (95% CI)
Age groups	15–59 years	NS	1
	60–69 years	NS	1.47 (1.01 to 1.98)
	70 years or over	NS	1.87 (1.37 to 2.58)
Gender	Male	1	1
	Female	1.79 (1.32 to 2.42)	1.37 (1.01 to 1.86)
Self-rated health	Very good/good	1	1
	Fair/poor/very poor	1.69 (1.16 to 2.47)	2.24 (1.62 to 3.11)
Limitations for usual activities	No	NS	1
	Yes	NS	1.56 (1.11 to 2.18)
Use of pain medication	No	1	1
	Yes	2.06 (1.49 to 2.82)	2.00 (1.50 to 2.67)
Mental disorder	No	1	NS
	Yes	1.48 (1.04 to 2.13)	NS
Migraine or frequent headache	No	1	1
	Yes	1.91 (1.23 to 2.97)	1.59 (1.04 to 2.56)
Body mass index	<25	NS	1
	25–29.9	NS	NS
	≥30	NS	1.33 (1.17 to 1.53)
Concomitant chronic neck pain	No	NIM	1
	Yes	NIM	10.46 (7.72 to 14.17)
Concomitant chronic low back pain	No	1	NIM
	Yes	10.46 (7.72 to 14.17)	NIM

NS: not significant. NIFM: not included in the model. OR: odds ratios estimated using multivariable unconditional logistic regression. CI: confidence interval.
